# Breast Cancer Therapy: The Potential Role of Mesenchymal Stem Cells in Translational Biomedical Research

**DOI:** 10.3390/biomedicines10051179

**Published:** 2022-05-19

**Authors:** Pietro Gentile

**Affiliations:** 1 Department of Surgical Science, “Tor Vergata” University, 00133 Rome, Italy; pietrogentile2004@libero.it; Tel.: +39-3388-5154-79; 2 Academy of International Regenerative Medicine & Surgery Societies (AIRMESS), 1201 Geneva, Switzerland

**Keywords:** breast cancer, breast cancer and mesenchymal stem cells, breast cancer therapy and stem cells, regenerative plastic surgery, plastic surgery

## Abstract

The potential role of mesenchymal stem cells (MSCs) in the treatment of metastatic cancers, including breast cancer, has been investigated for many years leading to encouraging results. The role of fat grafting and the related adipose-derived mesenchymal stem cells (AD-MSCs) has been detailed and described for breast reconstruction purposes confirming the safety of AD-MSCs. MSCs have great potential for delivering anticancer agents, suicide genes, and oncolytic viruses to tumors. Currently, many studies have focused on the products of MSCs, including extracellular vesicles (EVs), as a cell-free therapy. This work aimed to review and discuss the current knowledge on MSCs and their EVs in breast cancer therapy.

## 1. Introduction

Breast cancer (BC) is estimated to be responsible for 19 million disability-adjusted life years (DALYs) cases in women [1]. The survival rate in developed countries is high, while the number of deaths per affected woman is higher in low-income as well as middle-income countries [1]. The number of newly diagnosed BC cases in 2020 was about 2.3 million, as reported by the GLOBOCAN 2020 data [2]. According to current studies, this number could increase to 2.7 by 2030 [2]. Globally, BC’s mortality-to-incidence ratio (MIR) was 0.3 in 2020 [3]. The MIR shows a five-year survival rate in cancers [4]. From a biomolecular perspective, the expression of three molecules, estrogen receptor alpha (ERα), progesterone receptor (PR), and epidermal growth factor 2 receptor (ERBB2, formerly known as HER2 or HER2/neu), is important in the diagnosis, classification, and targeting of breast cancer [5]. ERα is expressed in 70% of invasive BC cases. The activation of ERα by estrogen induces oncogenic pathways in pro-cancer cells. In addition, PR expression is closely related to the ERα signaling pathway [6]. The molecule ERBB2 is overexpressed in 20% of BC cases [7]. Anti-ERBB2 therapy is a method of choice in the treatment of such invasive cases [8]. In triple-negative BC (T-n-BC), none of these three markers are expressed in the tumor. The molecule ERBB2 is responsible for 15% of BC cases and has the lowest survival rate; however, the specific molecular pathophysiology of this subtype still remains unclear [9].

The treatment of non-invasive BC is based on the removal of the tumor from the breast and treatment for the prevention of cancer metastasis. The use of trastuzumab anti-ERBB2 along with chemotherapy is also useful for the treatment of ERBB2-positive BC. In T-n-BC, chemotherapy is the method of choice. For metastatic BC (m-BC), the goal of therapy is to increase life and alleviate the symptoms of the disease. A combination of surgery, chemotherapy, adjuvant therapy, and targeted therapy (for example anti-ERBB2 antibody) is used for such patients; however, m-BC is mainly incurable, and the current treatments have undesirable consequences [10]. Therefore, many scientists have focused on mesenchymal stem cells (MSCs) and their products, such as exosomes, for the treatment of metastatic cancers, including m-BC. It has also been possible to engineer stem cells (SCs) to increase the efficiency of cancer therapy [11].

## 2. Role of MSCs on Breast Cancer

SCs are recognized for their ability to proliferate (self-renewal) and differentiate. MSCs are in almost all mammalian tissues including bone marrow, dental pulp, fat tissue, cord blood, amniotic fluid, and so on. They are responsible for tissue repair and regeneration during tissue/organ damage [12]. They have immunomodulatory properties, which are mediated through the expression of co-stimulatory molecules, allowing them to regulate cytokine secretion and immune responses. One of the main advantages of MSCs is that they can be used in an autologous manner. Therefore, MSCs have been investigated in cell therapy for human diseases for many years [13]. MSCs can migrate and home in on damaged sites, including tumor microenvironments. The migration and homing of MSCs are induced by cytokines and chemokines and are mediated through the expression of matrix metalloproteases (MMPs) [14]. Toll-like receptors (TLRs) are important for the function of MSCs in the tumor microenvironment. The recognition of antigens by TLR4 leads to the polarization of MSCs into type 1 (MSCs1), while TLR3 signaling stimulates the formation of MSCs2. MSCs1 is believed to possess antitumor effects, while MSCs2 inhibits tumor growth and metastasis [15]. MSCs’ functions, both antitumor and tumor-promoting activities, are mediated through cell–cell contact and paracrine effects. Paracrine effects have a predominant role in MSCs’ functions and are mainly attributed to extracellular vesicles (EVs) secreted by MSCs [16]. MSCs have a high tropism to tumor sites; therefore, many attempts have been performed to investigate the therapeutic role of MSCs on human tumors, including BC. However, studies have shown that MSCs have a dual role in BC. Some studies, both in vitro and in vivo, have shown that MSCs promote tumor progression by inducing cell growth, proliferation, metastasis, epithelial-to-mesenchymal transition (EMT), and drug resistance [17,18,19,20,21,22]. However, some other studies revealed that MSCs have antitumor activities by suppressing the cell cycle, inhibiting cell proliferation, inducing apoptosis, and promoting immune cell infiltration [23,24,25].

Many factors may be responsible for the discrepancies in the results of the previous studies; various cancer cells with different origins, the variability of stem cell donors, experimental conditions such as time and dose of injection, and the presence or absence of other treatment modalities could have affected the results [11,26]. Moreover, the number of tumor cells might be an important factor. It is believed that MSCs exert antitumor effects when the number of tumor cells is low and have tumor-promoting effects when the number of tumor cells is high [26]. These contradictory results have limited the use of MSCs for cancer treatment; however, technical improvements along with increasing knowledge about both tumors and stem cells have opened new lines of research in MSC-based BC therapy.

## 3. Engineered MSCs on Breast Cancer Models

### 3.1. Genetically Engineered MSCs

While MSCs have a dual role in BC, scientists have, however, evaluated the role of engineered MSCs in BC therapy, thanks to the introduction of therapeutic genes in MSCs (tumor suppressor genes, suicide genes, proapoptotic genes, and genes encoding cytokines and other immune activations genes) to inhibit tumor growth, induce apoptosis, and activate immune responses. Viral and non-viral methods have been used for the MSCs’ transfection of a therapeutic transgene. Non-viral methods have included electroporation or the use of liposomes and cationic polymers. However, non-viral methods had low efficiency and led to the transient expression of the desired transgene. Viral methods were based on genetically engineered viruses, such as lentiviruses, retroviruses, adenoviruses, adeno-associated viruses (AAVs), and other related viruses. These methods were more efficient to introduce transgenes into MSCs [11].

Amara et al. [27] used MSCs as carriers to deliver the suicide gene CYP2B6TM-RED (a mutant of CYP2B6 with NADPH cytochrome P450 reductase) into the tumor site. MSCs were transduced by a lentiviral vector. Their results showed the stable expression of the suicide gene and subsequent bioactivation of cyclophosphamide (CPA) in the tumor. In vivo, the intra-tumoral injections of engineered MSCs in the mouse model of m-BC led to the elimination of tumors in one-third of animals, with no tumor recurrence after 6 months of treatment [27]. The result of this study showed the possible use of engineered MSCs in BC treatment. In another study, Cai et al. [28] used MSCs to deliver immune-apoptotin and HER2 genes into BC. Immune-apoptotin is an anti-HER2 single-chain antibody (scFv). The authors showed that engineered MSCs were able to secrete immune-apoptotin constantly for a prolonged time at the tumor site. More importantly, the secretion of immune-apoptotin induced cell apoptosis at the tumor site. It has also been shown that the use of immune-apoptotin-expressed MSCs was more effective than the protein form of immune-apoptotin in killing cancer cells. This could be due to the prolonged secretion of immune-apoptotin by MSCs and the tropism ability of MSCs to target tumor sites [28].

Immunotherapy is an effective strategy in cancer therapy. One of the approaches for the immunotherapy of cancers is to use MSCs transduced with cytokines and other immune components. Ling et al. [29] used IFN-β-overexpressing MSCs for the treatment of m-BC. While the use of IFN-β in cancer treatment was limited due to its short life, they showed that engineered MSCs were able to secrete IFN-β for a long time. The results showed that IFN-β induced apoptosis in cancer cells by inhibiting the STAT3 signaling pathway [29]. In addition, IL-18- and IL-12-overexpressing MSCs have been used for BC treatment in vitro and in experimental preclinical models, and the results were promising.

In both studies, the use of engineered MSCs led to the inhibition of cancer cell growth [25,30]. Altogether, the results of these studies have shown the potential of genetically engineered MSCs in the BC treatment.

### 3.2. MSC as a Carrier: Delivery of Anticancer Agents

Drug resistance is the main problem during the use of anticancer agents. Resistance occurs due to the long-term use and high concentration of anticancer drugs, which increases the cytotoxicity of anticancer therapy [31]. Insufficient selectivity of anticancer agents is responsible for the problem [32]. MSCs have high tropism and homing abilities for tumor sites, and, thanks to this capacity, it has been possible to load MSCs with anticancer agents and imaging factors for tumor-targeted delivery [33,34]. However, drug efflux from MSCs limits this approach to be used in cancer therapy. Some studies have tried to use nanotechnology to solve the problem. In this regard, Xu et al. [33] conjugated Doxorubicin (DOX) with light-responsive magnetic nanoparticles. MSCs were loaded with nanoparticles and administered systematically. The results showed the control released of DOX at the tumor site resulted in tumor growth inhibition [33]. In a study by Saulite et al. [34], MSCs loaded with nanoparticles were used for imaging BC cells in a 3D co-culture system. The results showed that MDA-MB-231 cells efficiently uptook quantum dots (QDs) in the 3D culture, allowing the imaging and monitoring of cancer cells in vitro [34]. In another study, Yao et al. [35] used nano drug-loaded MSCs for treatment in the BC lung metastasis model. They conjugated DOX with RGD polymer and introduced the drug into MSCs. Following the systemic injection, the nano drug-loaded MSCs homed in on the tumor site in the lung for a long time. The release of DOX at the tumor site induced cancer cell apoptosis, resulting in a reduction of the tumor size [35].

### 3.3. Delivery of Oncolytic Virus

Oncolytic viruses have been designed to specifically target cancer cells and initiate the apoptotic pathways. In addition, these viruses could be engineered to make cancer cells more susceptible to anticancer therapies, both chemotherapy and radiotherapy [36]. Oncolytic virotherapy changes the tumor microenvironment as induces cancer cell lysis and subsequently increases immune cells’ infiltration and cytokine production [37]. To date, several oncolytic viruses have been used during cancer therapy, which include recombinant herpes simplex virus type 1 (HSV-1), recombinant Newcastle disease virus (NDV), vesicular stomatitis virus (VSV), recombinant adenoviruses, Vaccinia virus [37,38]. The first oncolytic virus that was approved by the United States food and drug administration (US-FDA) in 2015 for the treatment of metastatic melanoma was Talimogene laherparepvec (T-VEC), a recombinant HSV-1. T-VEC was engineered in a way that proliferates selectively and expresses human granulocyte–macrophage colony-stimulating factor (GM-CSF) to activate immune cells [39]. Oncolytic viruses need to be delivered to the tumor sites. In some studies, MSCs have been used to systematically or non-systematically deliver oncolytic viruses for the treatment of various cancers and various degrees of success have been achieved [36,40,41,42]. In this approach, MSCs were transfected with oncolytic viruses as they have tropism to the tumor microenvironment. MSCs deliver the viruses at the tumor site and the released viruses transfect tumor cells [36].

**Figure 1:**
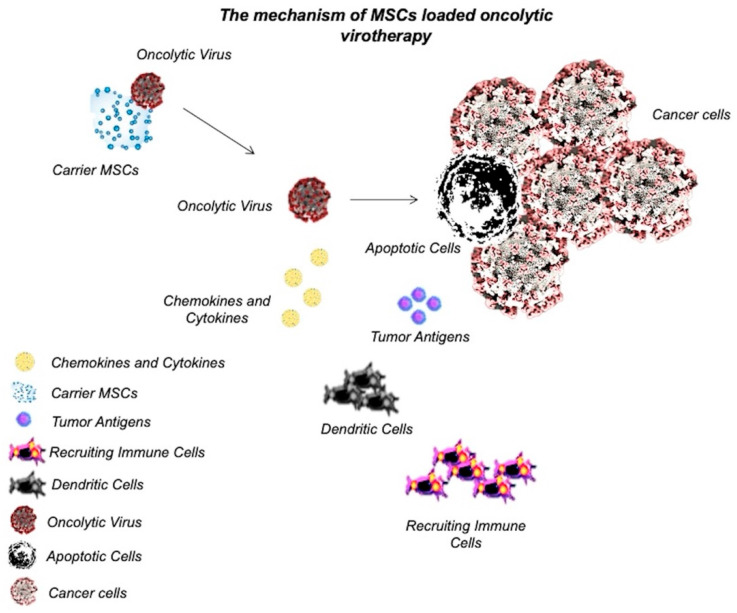
Mechanism of MSC-loaded oncolytic virotherapy.

Stoff-Khalili et al. [43] used MSCs to deliver conditionally replicating adenoviruses (CRAds) to lung metastasis in BC. To increase the tumor specificity, the CRAd virus was transfected with the E1A gene under the CXCR4 promoter (a tumor-selective promoter), allowing the expression of the target gene at tumor sites. Moreover, human adenovirus serotype 5 was incorporated into the virus to enhance tumor infectivity. The CRAd Ad5/3.CXCR4 displayed oncolytic activity on the MB-MDA-231 cancer cell line in vitro. The virus-loaded MSCs were systematically injected into a SCID mouse xenograft model. The results showed that engineered MSCs efficiently homed in on the tumor microenvironment and delivered CRAds into tumor cells. Systemically administered virus-loaded MSCs reduced the growth of tumors in the lung and improved the survival of mice [43]. Hakkarainen et al. [44] also used an adenovirus as an oncolytic virus and delivered the viruses to the tumor site by loading them into MSCs. In vitro results showed that the virus infectivity was enhanced by up to 11000-fold in heparan sulfate (HS) proteoglycan- and integrin-targeted viruses rather than adenovirus serotype 5 (Ad5). In vivo results also showed that an intravenous injection of virus-loaded MSCs increased the tumor homing of a virus and enhanced survival in the mouse model of lung cancer and BC [44]. Although few studies have used MSCs as a carrier of oncolytic viruses in BC therapy, the results were promising. Moreover, delivery of oncolytic viruses to the tumor microenvironment through MSCs has shown various degrees of success in the treatment of other types of tumors, such as glioma [45], neuroblastoma [46], ovarian cancer [47], colon cancer (CRC) [48], and so on. Therefore, it could be considered an effective BC therapy for future studies. The mechanism of MSC-loaded oncolytic virotherapy has been graphically described in Figure 1.

## 4. MSC-Derived EVs on Breast Cancer

### 4.1. Extracellular Vesicles

The functions of MSCs are mediated not only by direct cell–cell contact but also through paracrine effects. In the latter, the secretome of MSCs is involved. Secretome contains a variety of molecules as well as ultrastructures, such as extracellular vesicles (EVs) [49]. EVs have been declared to be produced in almost all cell types, both in physiological and pathological situations. In mammalian cells, different biological substances, including RNAs, proteins, and lipids, are carried to the target cell/tissue/organ and act as a part of the cellular communication [50]. EVs are lipid-bound nanoparticles that are like liposomes from a drug delivery perspective. EVs are classified into several classes, including exosomes, ectosomes, micro-vesicles (MVs), membrane vesicles, and apoptotic bodies. These subpopulations are heterogeneous and varied from each other in terms of size, content, production, and function. Among the subpopulations, exosomes have attracted much attention as they mediate many cellular processes and have a suitable size to be used in drug delivery approaches [50]. Exosomes are 40–100 nm nanoparticles in size, and their sucrose gradient density varies from 1.13 g/mL in B lymphocytes to 1.19 g/mL in the epithelial cells [51]. Several inductions are involved in exosome biogenesis, including cellular stress, irradiation, hypoxia, or starvation, which have been shown to increase exosome production [52]. In the cell source, multivesicular bodies (MVBs) are formed in the endosomal pathway. These MVBs contain intraluminal vesicles (ILVs), which are further matured into exosomes through the diffusion of some specific proteins. The exosomes are released into the extracellular space as MVBs are fused to the cellular membrane [53,54]. The exosomes represented a spheroid in a solution under transmission electron microscopy [55]. Recent studies have demonstrated the effect of EVs in various physiological and pathological conditions, such as immune responses, viral pathogenicity, cardiovascular diseases, central nervous system-related diseases, and cancer progression [52,56]. Moreover, EVs from immunomodulatory cells have shown promising therapeutic properties in the treatment of diseases such as cancers, inflammatory diseases, and so on [57,58,59]. EVs have also provided many opportunities for efficient cargo delivery, in which different proteins, metabolites, and nucleic acids could be successfully delivered by exosomes into target cells [60]. Several studies have used EVs from either native or engineered MSCs as therapeutic options in BC therapy.

### 4.2. EVs Derived from MSCs on Breast Cancer

Several studies have used exosomes from MSCs to inhibit BC proliferation and progression. Casson et al. [61] showed that EVs derived from MSCs induced dormancy in the MCF-7 BC cell line. The results indicated that EVs inhibited the proliferation of MCF-7 cells in 2D and 3D cell cultures. Moreover, the genes involved in cell adhesion were upregulated in cancer cell lines, which showed the suppression of cancer cell migration [61]. Similar results were observed in the study by Sandiford et al. [62]. In another study, Li et al. [63] showed that EVs from MSCs conferred antitumor activity in BC mouse models. They isolated EVs from CD90low adipose-derived MSCs (CD90low AD-MSCs) and showed that the proliferation and migration of cancer cells were suppressed in vitro as well as in the BC mouse model [63]. Despite the results of these studies, Zhou et al. [64] showed that EVs derived from MSCs in a human umbilical cord (hUC-MSCs) increased the proliferation and migration of the MCF-7 BC cell line. They also showed that these EVs promoted EMT in cancer cells. The results showed that the promoting effects of EVs were mediated through the activation of the extracellular signal-regulated kinase (ERK) signaling pathway [64]. In another study, Khanh et al. [65] also showed that EVs derived from MSCs from diabetic patients enhanced the metastasis of BC cells in vitro. These contradictory results might be due to the dual roles of MSCs on cancers [65]. MSCs mediate their functions through cell–cell contact as well as paracrine signals. The paracrine signals are also mediated through EVs and other secretory molecules [66,67].

### 4.3. EVs Derived from Engineered MSCs on Breast Cancer

As mentioned above, engineered MSCs or EVs derived from MSCs have been used in the inhibition of BC cell proliferation and progression. In addition, it is possible to combine these two therapeutic approaches and evaluate the EVs from engineered MSCs on cancer cells. In an in vitro study, Altanerova et al. [68] transfected MSCs with a suicide gene and fused yeast cytosine deaminase::uracil phosphoribosyl transferase (yCD::UPRT). They showed that MSCs expressed the transfected gene in their EVs. The EVs were isolated and added to the MDA-MB-231 breast adenocarcinoma cell line. The results showed that EVs in the presence of the 5-fluorocytosine (5-FC) prodrug inhibited proliferation and apoptosis in cancer cells. Cytosine deaminase::uracil phosphoribosyl transferase expressed in the cytoplasm of target cells converted 5-FC to cytotoxic 5-fluorouracil (5-FU) [68].

In another study, O’Brien et al. [69] engineered MSCs to produce EVs enriched in miR-379. They transfected MSCs with a lentiviral vector containing miR-379 tumor suppressor and isolated miR-379-enriched EVs with normal morphology. Engineered MSCs and miR-379-enriched EVs were systematically administered into a BC mouse model. The results showed that engineered MSCs were not able to inhibit the proliferation and progression of cancer cells. However, when cell-free miR-379-enriched EVs were administered, the tumor size and mass were reduced. Moreover, the tumor was full of fluid following the administration of cell-free miR-379-enriched EVs; miR-379 is a tumor suppressor and its expression is decreased in BC patients [69]. It also suppresses the proliferation and progression of cancer cells and inhibits EMT [70].

### 4.4. EVs Derived from MSCs Carrying miR-Cargo in Chemoresistance and Dormant Breast Cancer: Limits and New Perspectives

Emerging evidence has shown the role of mesenchymal stem cell-derived exosome (MSC-Exo) in inducing the resistance of cancer cells to chemotherapy; however, it remains unclear whether the change in MSC-Exo in response to chemotherapy also contributes to chemoresistance. In a study by Luo et al. [71], the authors investigated the effect of a standard-of-care chemotherapeutic agent, doxorubicin (Dox), on MSC-Exo and its contribution to the development of Dox resistance in breast cancer cells (BCs).The authors found that the exosome secreted by the Dox-treated MSCs (Dt-MSC-Exo) induced a higher degree of Dox resistance in BCs when compared with non-treated MSC-Exo.

On the other point, dormant BC resurged as a metastatic disease after a long dormancy period in the bone marrow, where BCs interacted with MSCs. However, how early interactions between BCs and MSCs in the bone marrow microenvironment facilitate an adaptation to a quiescent state remains poorly understood.

The problem of dormant BC and its relationship with MSCs still needs to be deeply investigated. BC patients often develop metastatic disease years after resection of the primary tumor. The patients are asymptomatic because the disseminated cells appear to become dormant and are undetectable. As the proliferation of these cells is slowed, dormant cells are often unresponsive to traditional chemotherapies that exploit the rapid cell cycling of most cancer cells. For these reasons, in a study by Ono et al. [72], the researchers generated a bone marrow metastatic human breast cancer cell line (BM2) by tracking and isolating fluorescent-labeled MDA-MB-231 cells that disseminated to the bone marrow in mice. Coculturing BM2 cells with bone marrow mesenchymal stem cells (BM-MSCs) isolated from human donors revealed that BM-MSCs suppressed the proliferation of BM2 cells and decreased the abundance of stem cell-like surface markers, inhibited their invasion through Matrigel Transwells, and decreased their sensitivity to docetaxel, a common chemotherapy agent. Acquisition of these dormant phenotypes in BM2 cells was also observed by culturing the cells in a BM-MSC-conditioned medium or with exosomes isolated from BM-MSC cultures, which were taken up by BM2 cells. Among various microRNAs (miRNAs) increased in BM-MSC-derived exosomes compared with those from adult fibroblasts, overexpression of miR-23b in BM2 cells induced dormant phenotypes through the suppression of a target gene, MARCKS, which encodes a protein that promotes cell cycling and motility. Metastatic breast cancer cells in patient bone marrow increased miR-23b and decreased MARCKS expression. Together, these findings suggested that the exosomal transfer of miRNAs from the bone marrow might promote breast cancer cell dormancy in a metastatic niche [72].

## 5. Adipose-Derived Mesenchymal Stem Cells (AD-MSCs) and Fat Grafting in Breast Cancer and Related Outcomes

In the last few years, surgical procedures in BC outcomes were deeply modified with a gradual shifting to less invasive strategies based on autologous fat grafting (FG) [73].

This last interesting strategy of breast reconstruction based on minimal manipulation of fat tissue via centrifugation, filtration, or enzymatic digestion using human collagenases has been used also in breast augmentation for aesthetical purposes with excellent outcomes [74]. Previously the FG was used also for others soft tissue defects [75,76]

A recent study compared breast remodeling results obtained in patients suffering from breast hypoplasia, treated with definitive implants (DI), with those obtained in patients treated with FG enriched with adipose-derived mesenchymal stem cells (AD-MSCs). The influence of breast and chest deformities (TB, volume, nipple–areola complex [NAC] asymmetry, pectus excavatum, and carinatum) in the reconstructive outcome was also analyzed [77]. The study confirmed the safety and effectiveness of DI and AD-MSCs-enhanced FG in the treated case series, showing that FG allowed for decreased scar burden with natural aesthetic results.

The main limits of FG is resorption and the controversial breast cancer relationship in obese patients. The most important and recent studies on breast remodeling procedures performed with FG described a 58% maintenance of the fat volume after 3 years when FG was enriched with AD-MSCs, compared with FG without the addition of AD-MSCs, which showed a 29% maintenance [74,77]

The techniques based on FG (not-enriched FG or FG enriched with AD-MSCs) did not represent a significant risk factor for tumor recurrences, as confirmed in recent clinical trials [73,74,75,76,77,78]. Additionally, a recent innovative strategy during conservative mastectomies and pre-pectoral breast reconstructions, based on titanium mesh, which could be used also in combination with FG, has been described, confirming its oncological safety [79].

Even though the major cellular burden in BC is constituted by the bulk tumor cells, another cell subpopulation named cancer stem cells (CSCs) has been described [80]. The latter have stem features, a self-renewal capacity, and the ability to regenerate the bulk tumor cells. CSCs have been described in several cancer types, but breast cancer stem cells (BCSCs) were among the first to be identified and characterized [80]. Many dysregulated pathways in BCSCs are involved in the epithelial–mesenchymal transition (EMT) and are found upregulated in circulating tumor cells (CTCs), another important cancer cell subpopulation that shed into the vasculature and disseminated along the body to give metastases. Conventional therapies fail at eliminating BCSCs because of their quiescent state, which gives them therapy resistance. Markers useful for BCSC identification could also be possible therapeutic methods against BCSCs [80]. New approaches in drug delivery combined with gene targeting, immunomodulatory, and cell-based therapies could be promising tools for developing effective CSC-targeted drugs against BC [80].

As reported, the beneficial effects of FG during breast reconstruction have been amplified by the enrichment with human AD-MSCs. The major concern about the AD-MSC enrichment during breast reconstruction for BC outcomes depends on their potential ability to release growth factors and hormones that can promote proliferation of residual or quiescent cancer cells, with the risk of de novo cancer development or recurrence. The recent description that adult stem cells primed in vitro may be a vehicle for anti-cancer drug delivery offers a new vision concerning the role of AD-MSCs in breast reconstruction after cancer removal [81]. Paclitaxel (PTX) is a chemotherapeutic agent acting as a microtubule-stabilizing drug inhibiting cancer cell mitotic activity. A recent study [81] optimized PTX loading and release in cultured AD-MSCs and then analyzed the effects of PTX-loaded AD-MSCs and their conditioned medium on CG5 BC survival, proliferation, and apoptosis in vitro, and in a CG5 xenograft in vivo [81]. Interestingly, PTX-loaded AD-MSCs in a co-cultured and conditioned medium alone inhibited CG5 cell proliferation and survival in vitro and xenograft tumor growth in vivo [81]. The antitumor effect of PTX-loaded AD-MSCs may offer a new perspective concerning the use of AD-MSCs during breast reconstruction, and could become an additional local preventive chemotherapeutic agent against tumor recurrence.

## 6. Conclusions and Future Perspective

MSCs have been shown to have a dual role in BC, which could be due to the variability in cell sources, individual donors, and experimental conditions. However, engineered MSCs have been successfully used in the treatment of BC in vitro and in vivo with promising results. The MSCs may be genetically modified to overexpress beneficial genes including cytokines (to activate immune systems) or suicide genes (to induce apoptosis in cancer cells). Thanks to the high ability to home in on tumor sites expressed by MSCs, several attempts have been applied to deliver anticancer agents to tumors using MSCs. In this regard, MSCs delivered DOX or oncolytic viruses to the tumor site and subsequently inhibited tumor growth and induced apoptosis. MSCs mediated their function through cell–cell interactions and their EVs. Like native MSCs, EVs from native MSCs have also been shown to have a dual role in cancer therapy. However, EVs from engineered MSCs allowed efficient BC therapy. EVs derived from MSCs transfected with a suicide gene or miRNA (miR-379) were able to inhibit growth and induce apoptosis in tumor models. Despite these new perspectives, several limits of EVs have been identified:

- they induce resistance of cancer cells to chemotherapy;
- the interactions between BCs and MSCs in the bone marrow facilitates adaptation to a quiescent state.

In light of the limits and the therapeutic potential of EVs derived from engineered MSCs reported here by only a few studies in vitro and in vivo, the author suggests that more research is needed to confirm these preliminary outcomes. This need for further research is also based on current needs to advance the therapeutic potential and/or practical implementation of the MSC secretome.

## Data Availability

Not applicable.
